# The Role of Fish Skin Xenografts in Healing Complex Wounds: A Brief Case Report

**DOI:** 10.7759/cureus.56156

**Published:** 2024-03-14

**Authors:** Camryn Daidone, Naved Salim, Leslie Smith, Ahsan Raza

**Affiliations:** 1 Surgery, Edward Via College of Osteopathic Medicine, Shreveport, USA; 2 Medicine, Edward Via College of Osteopathic Medicine, Shreveport, USA; 3 General Surgery, Rapides Regional Medical Center, Alexandria, USA

**Keywords:** necrotizing wound, fish skin graft, nonhealing wound, xenografts, advanced wound care, general surgery

## Abstract

Non-healing wounds profoundly impact patient quality of life and present a significant financial burden. The Kerecis™ fish skin xenograft is a decellularized skin matrix that has been introduced to treat complicated wounds. The objective of this presentation is to highlight the use of fish skin xenograft in the treatment of a complex right flank wound with stool contamination, necrotizing soft tissue infection due to perforated colon cancer, and sepsis. This presentation follows the wound healing for 28 days following the operation and demonstrates the efficacy of fish skin xenografts in improved wound healing. A 61-year-old female with a past medical history of colon cancer and recent chemotherapy treatment presented with colon perforation causing right flank cellulitis and sepsis with necrotic abdominal wall tissue extending into the hip joint. She was taken for an emergent exploratory laparotomy, drainage of abdominal and retroperitoneal abscesses, open right hemicolectomy, diverting ileostomy, abdominal washout, intra-abdominal omental patch, placement of Strattice mesh, and debridement of necrotizing soft tissue infection of the right flank. After extensive debridement of her 15x10cmx5cm deep wound and placement of a Kerecis™ fish skin xenograft, the wound had completely healed with excellent granulation tissue, and the patient was scheduled for placement of a skin graft 28 days following the initial procedure. The results after xenograft application were outstanding, supporting the use of polyunsaturated fatty acid (PUFA) based xenografts in wound treatment due to their anti-inflammatory and angiogenic properties. This is definitely an option that needs to be considered in expediting the healing process for complex wounds.

## Introduction

Chronic non-healing wounds present a substantial impact on the quality of life of approximately 2.5% of the United States population and pose a significant financial burden. The estimated Medicare cost projection for wound care was between $28.1 and $96.8 billion in 2020 alone [1]. Of these costs, up to $35.8 billion were associated with outpatient wound care costs. With the increase in the incidence of chronic non-healing and complex acute wounds due to conditions such as diabetes, pressure ulcers, and vascular deficits, it is becoming increasingly important to develop a gold-standard treatment and agents to facilitate effective wound healing [1].

The Kerecis™ fish skin xenograft is a decellularized fish skin matrix derived from North Atlantic Cod Fish (Gadus Morhua). Due to patented processing (cell lysis via osmotic mechanism), the skins retain a highly porous microstructure that is homogenous to human dermis, noncellular proteins, and polyunsaturated fatty acids (PUFAs). Kerecis fish skin xenograft products have been introduced for the treatment of complicated wounds such as diabetic wounds, traumatic wounds, partial-thickness burns, acute surgical incisions, and necrotic wounds [2,3]. Due to the known anti-inflammatory properties of PUFAs, fish skin xenografts have shown promise in wound healing when compared to traditional wound management techniques such as autografts using the patient's tissue, allografts of donated human tissues such as dehydrated human amnio-chorionic membrane, or management without the use of tissue grafts with frequent debridement and dressing changes [4,5]. PUFAs possess anti-inflammatory properties, are able to mitigate cytokine signaling, and may decrease the risk of bacterial colonization in the context of wound healing.

The objective of this presentation is to highlight the use of fish skin xenograft in the treatment of a particularly complex right flank wound which included stool-contamination, necrotizing soft tissue infection due to perforated colon cancer, and sepsis. This presentation follows the wound healing through 28 days after the operation and demonstrates the efficacy of omega-3 fish xenografts in improving the healing of complex wounds.

## Case presentation

**Figure 1:**
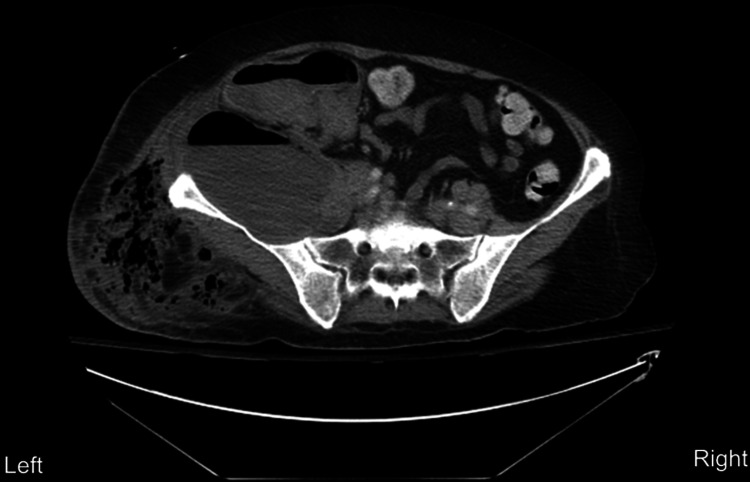
CT scan taken during emergency department admission. The scan was remarkable for a large retroperitoneal abscess with lateral extension through the lower lateral abdominal wall musculature into the subcutaneous tissues. Area of retroperitoneal abscess posterior to the cecum and along the iliac is muscle measures approximately 15 cm in maximal length.

A 61-year-old female presented to the emergency department with a two-week history of pain. She presented afebrile (97.7F) and normotensive (120/65) with profound tenderness and crepitus appreciated on the right flank with a small area of purulence and necrotic tissue. Initial studies were significant for a white blood cell count of 9.4. The patient was taken for an emergent exploratory laparotomy, drainage of abdominal and retroperitoneal abscess, open right hemicolectomy with diverting ileostomy, abdominal washout, intra-abdominal omental patch, placement of Reconstructive Tissue Matrix (Strattice) mesh for flank hernia prevention, and debridement of necrotizing soft tissue infection of the right flank. Figure 1 shows a CT scan of the region prior to surgery.

**Figure 2:**
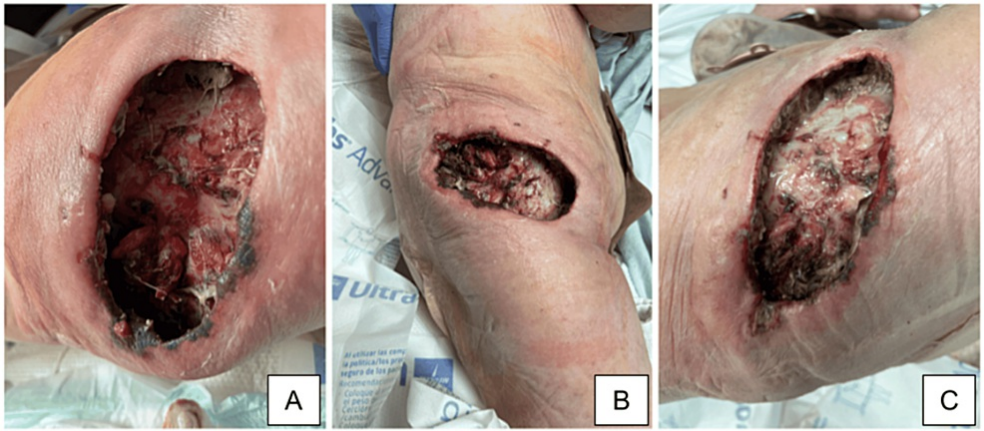
Right flank wound 2 days postoperative following wound debridement. The dimensions of this wound were 15cm x 10cm x 5cm deep.

Necrotic abdominal wall tissue and invasion of the hip joint were noted during surgery. There was stool contamination of the wound necessitating extensive debridement. The wound was 15cm x 10cm x 5cm deep and was initially debrided and washed with Dakin’s Solution (Century Pharmaceuticals, Inc) sodium hypochlorite antiseptic and placed on a Dakin’s wet-dry bandage (Century Pharmaceuticals, Inc). Figures 2A-2C show this wound two days after washing and debridement. The patient consented to the use of clinical information and the included images in a case report and no personal health information was included to protect patient anonymity.

**Figure 3:**
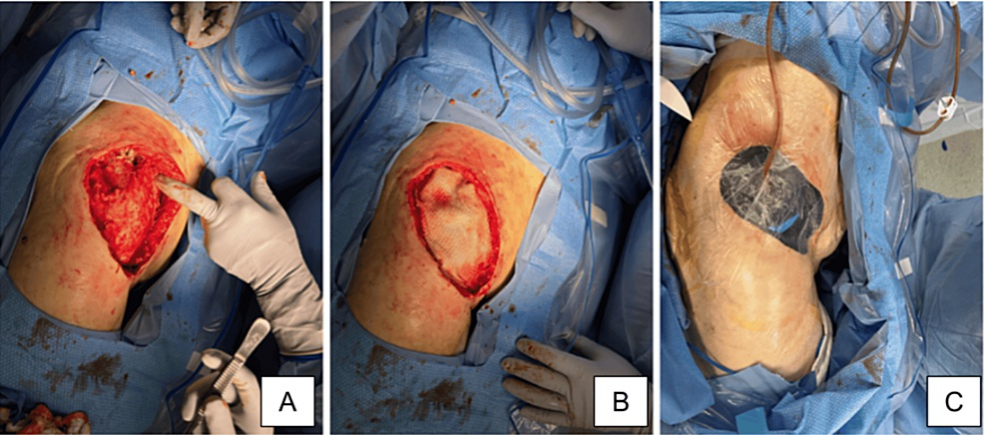
Further debridement (A), placement of xenograft (B) and closure with wound VAC (C). Dimensions of the wound were 17cm x 10cm x 4cm.

**Figure 4:**
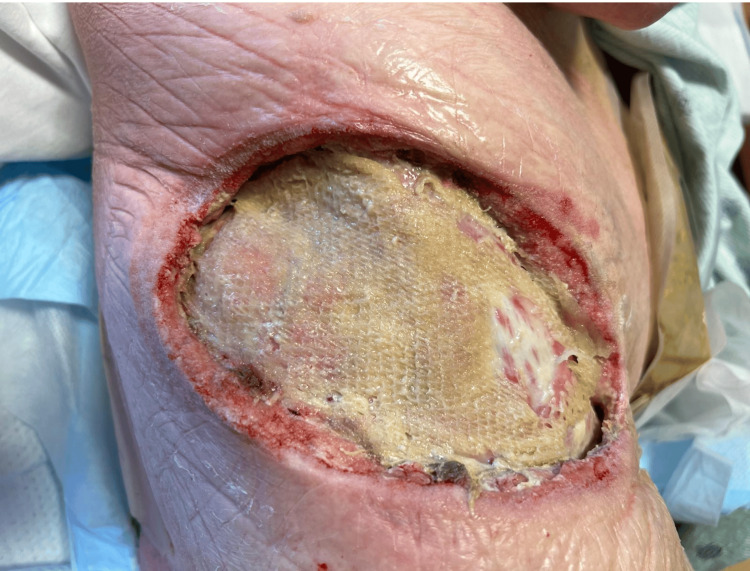
Wound five days following xenograft placement. No signs of cellulitis, no tunneling, some sloughing of tissue.

**Figure 5:**
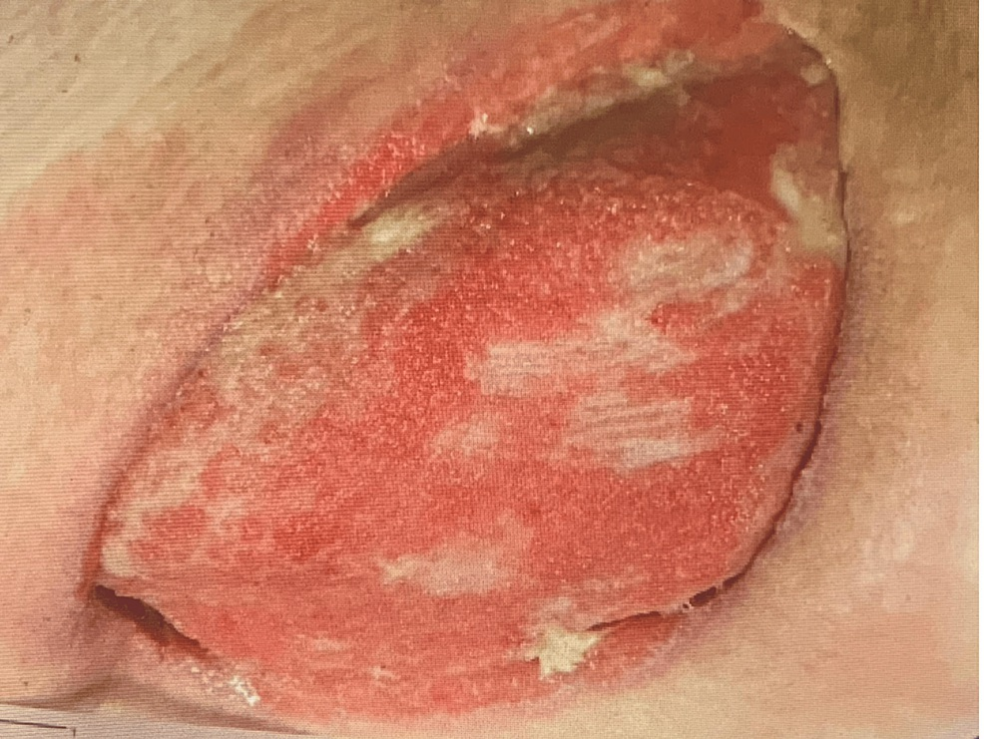
Wound approximately one week following xenograft placement. The wound appears healthy with granulation tissue present and no tunneling. At this point, the patient was discharged home with a wound VAC.

**Figure 6:**
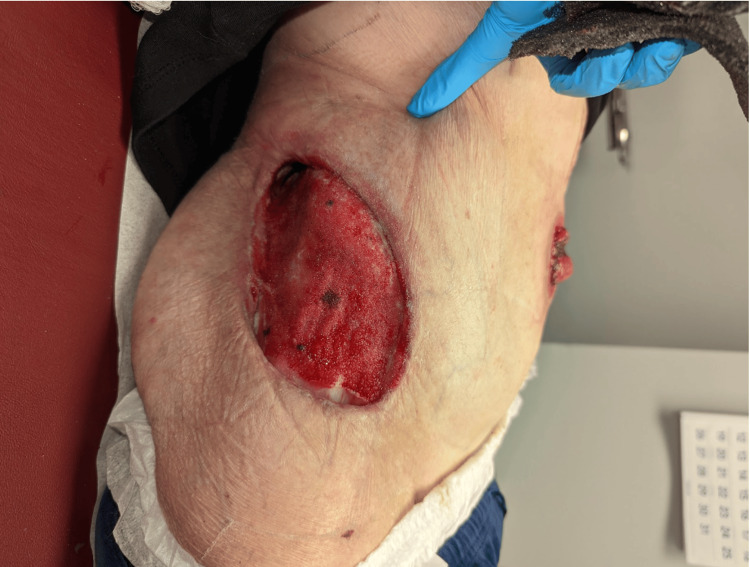
Wound on postoperative day 28 follow-up visit, the wound is healed with substantial granulation tissue and the patient is scheduled for placement of a skin graft.

Two days post-operation, further debridement, and application of a Kerecis™ xenograft with a wound vacuum-assisted closure (VAC) was completed (Figures 3A-3C). The wound was followed over the next week in the hospital with periodic VAC changes as shown in Figures 4-6, until the patient was discharged home and followed by home health for wound care. On postoperative day 28, a follow-up visit revealed that the wound had healed well with substantial granulation tissue present (Figure 6). The patient was then scheduled for placement of a skin graft.

## Discussion

Kerecis™ fish skin xenografts are FDA-approved for treating chronic and acute surgical wounds [6,7]. The product is an acellular dermal matrix harvested from Icelandic cod with a porous microstructure similar to that of human skin. Characteristics of the xenograft include bacterial resistance, rapid cellular migration and proliferation, and inflammatory cytokine mitigation due to the abundance of PUFAs in the matrix [8]. This presentation noted excellent wound healing after one application of the Kerecis™ product for a wound complicated initially by stool contamination and necrotic tissue. Additionally, this patient had recently completed chemotherapy for colon cancer and was septic upon presentation to the emergency department, both of which would predispose this patient to impairments in wound healing [9].

Previous studies support these findings and have demonstrated the efficacy of the use of intact fish skin xenografts for the treatment of necrotizing fasciitis of the leg and the superiority of fish skin graft (FSG) over xenograft alternatives such as fetal bovine dermis (FBD) in treating deep partial thickness burns [4,10].

Despite evidence suggesting the benefits of fish skin xenografts, one substantial drawback of the use of the Kerecis™ product is the increased associated cost. This may be especially problematic in areas with low healthcare coverage. However, one study conducted a cost analysis of using the Kerecis™ product against annual healthcare costs and found that annual costs were actually decreased due to faster wound healing [11]. With wound care posing a significant financial burden nationally, the cost associated with fish skin xenografts may eventually offset the costs of chronic wound care [1].

Future studies with larger sample sizes are needed to further support the efficacy of the Kerecis™ product in treating necrotizing wounds and comparisons to other xenograft alternatives. Future research should investigate the use of fish skin xenografts in various types of wounds such as pressure ulcers and those complicated by diabetes, immunodeficiency, or vascular insufficiency as well as the potential contraindications, complications, and costs associated with these products.

## Conclusions

Application of the Kerecis™ Fish Skin xenograft yielded outstanding results in wound healing for this patient with a wound complicated by necrotizing tissue, stool contamination and immunodeficiency from recent chemotherapy use. This supports the anti-inflammatory and angiogenic properties of the product and efficacy in healing complex wounds. Our successful application of fish skin xenografts in this patient case further supports the use of these grafts for a wider range of injuries including superficial burns or pilonidal abscesses healing through secondary intention. This is certainly an option that needs to be considered in treating difficult wounds in patients who are already immunocompromised. Future research should investigate the various indications of fish skin xenografts as well as the potential contraindications, complications, and costs associated with these products.
